# Supplementary figures and images for: Discoidin Domain Receptor 1 (DDR1) tyrosine kinase is upregulated in PKD kidneys but does not play a role in the pathogenesis of polycystic kidney disease
Source: PLoS One. 2019 Jul 1;14(7):e0211670. doi: 10.1371/journal.pone.0211670 (PMC6602183; doi:10.1371/journal.pone.0211670)

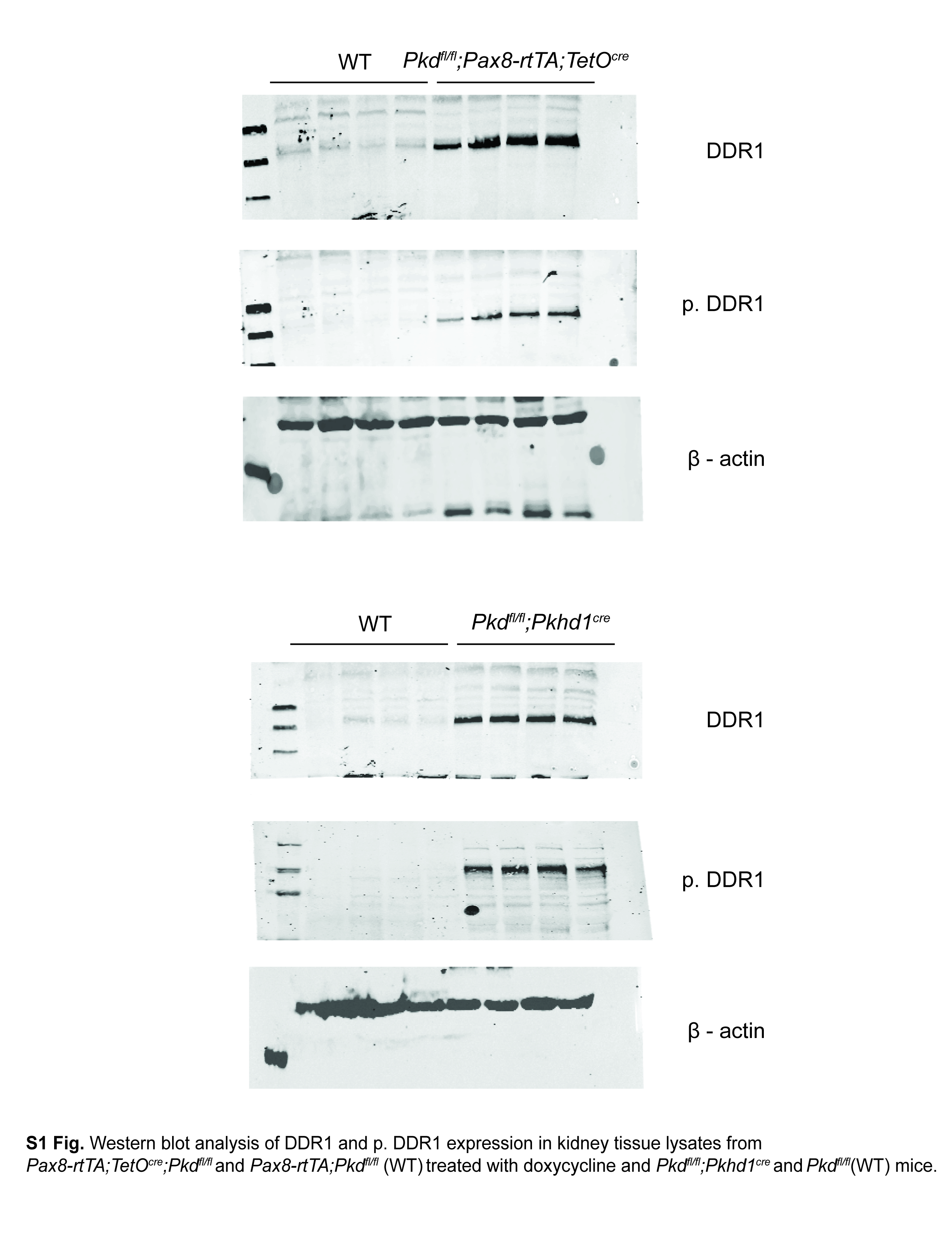

Supplement: S1 Fig — (TIF) [file pone.0211670.s001.tif]

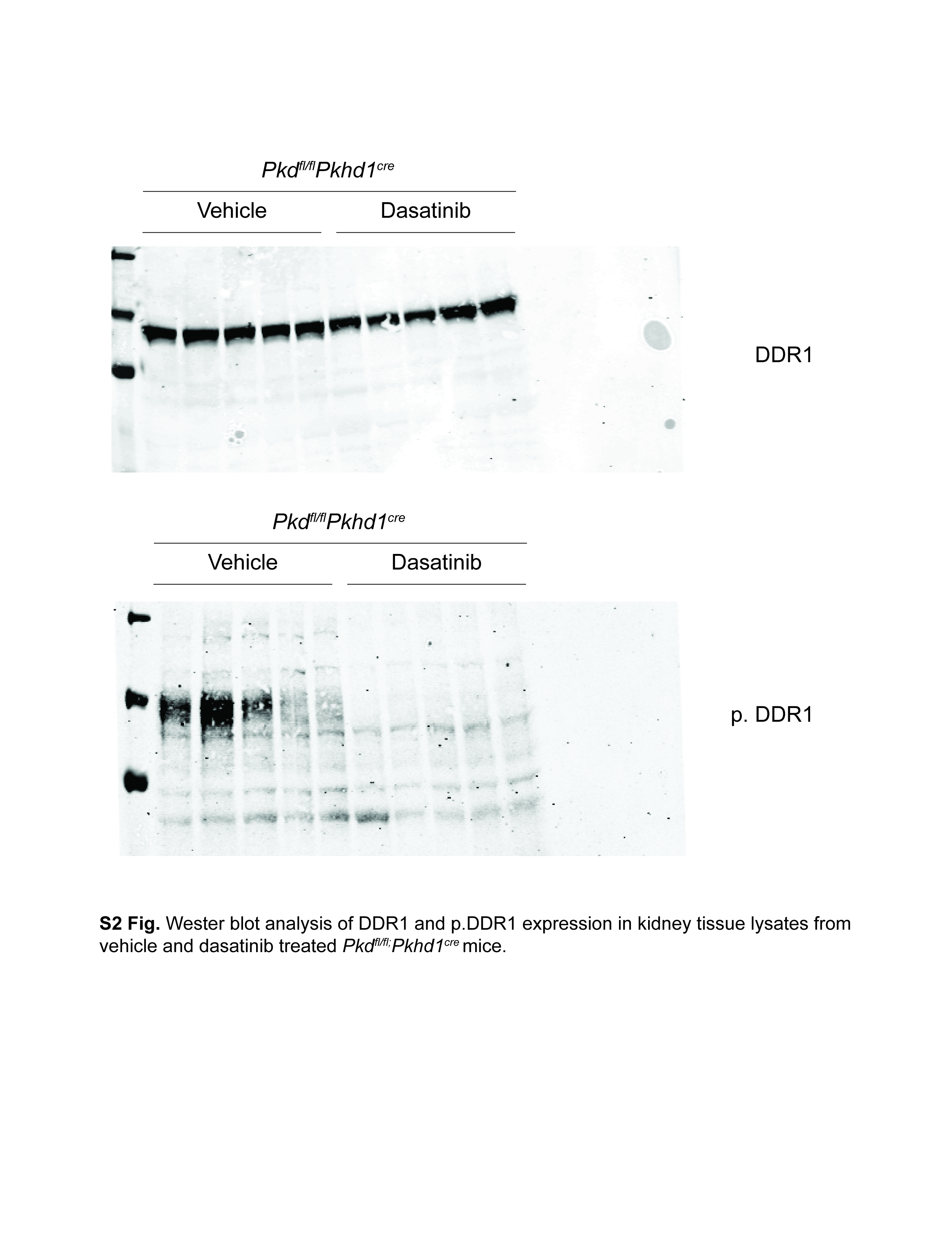

Supplement: S2 Fig — (TIF) [file pone.0211670.s002.tif]

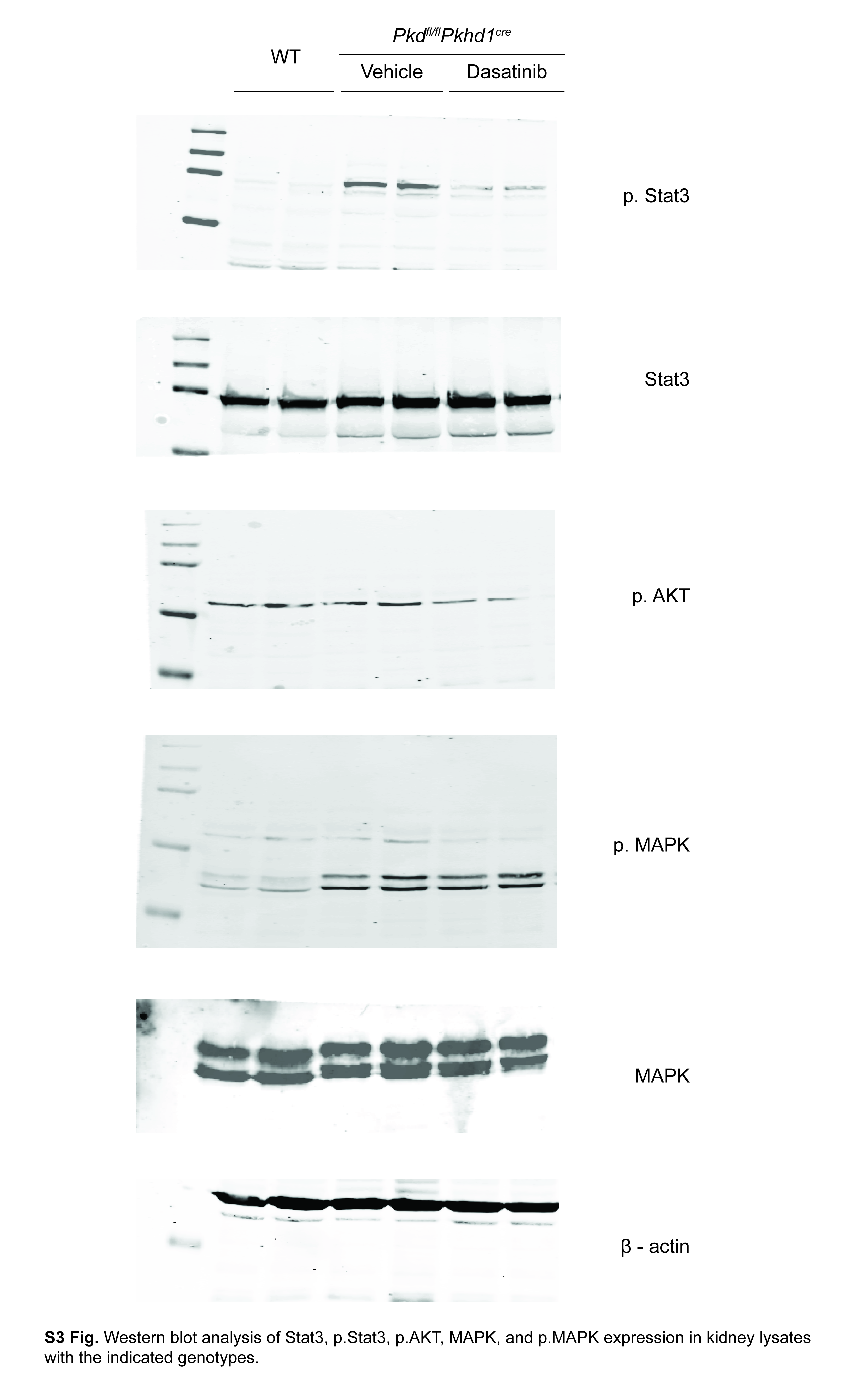

Supplement: S3 Fig — (TIF) [file pone.0211670.s003.tif]

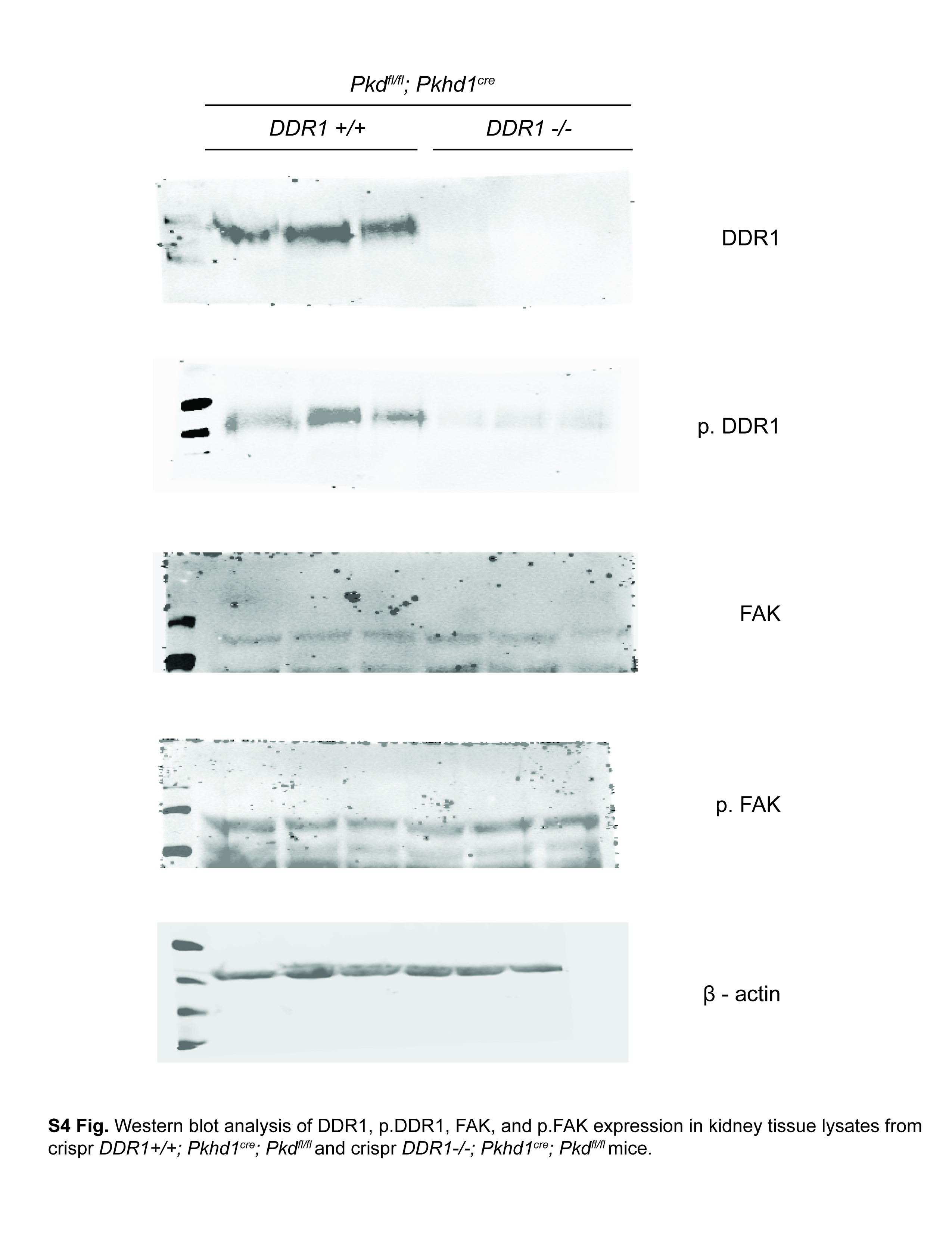

Supplement: S4 Fig — (TIF) [file pone.0211670.s004.tif]

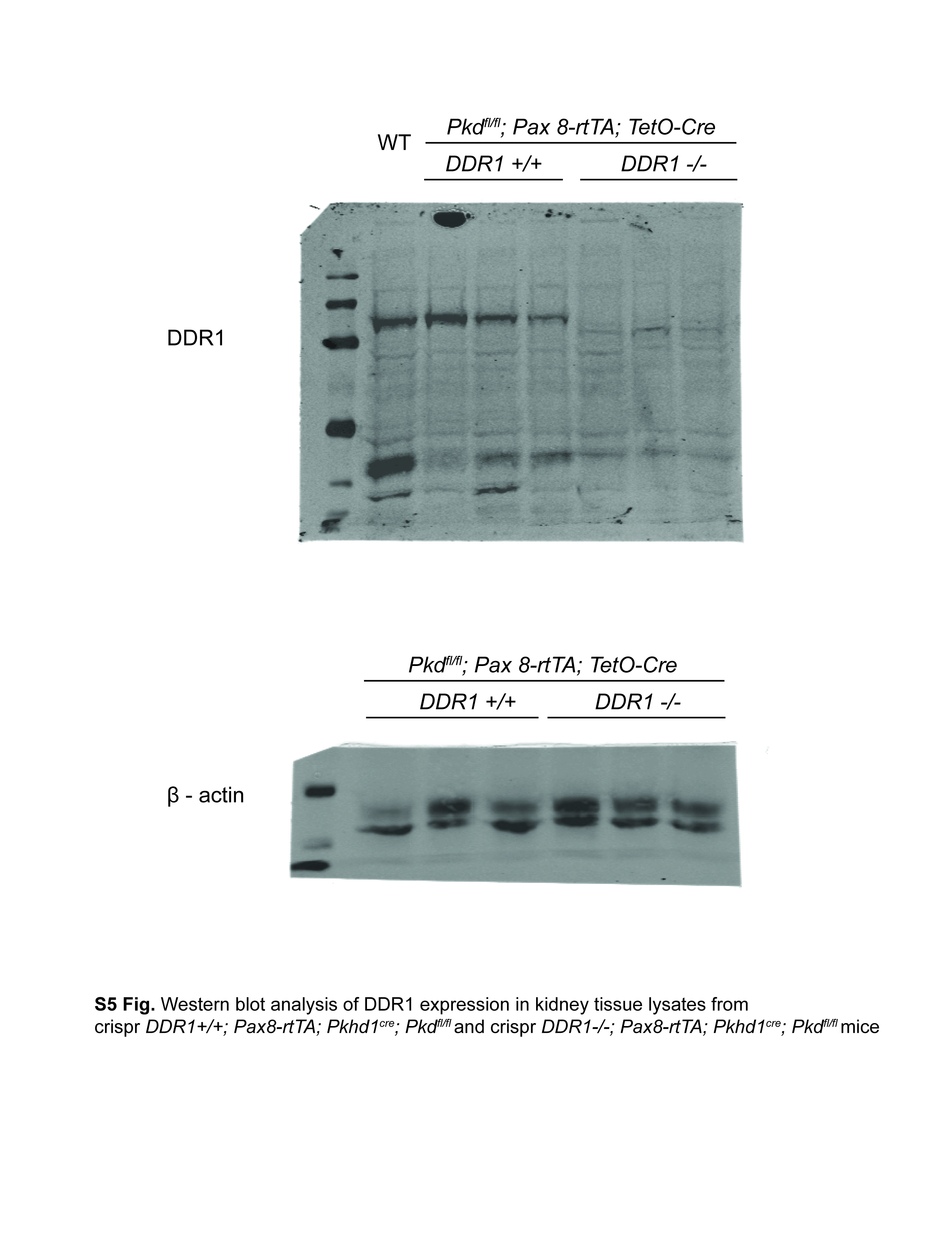

Supplement: S5 Fig — (TIF) [file pone.0211670.s005.tif]
